# Cementless anatomical prosthesis for the treatment of 3-part and 4-part proximal humerus fractures: cadaver study and prospective clinical study with minimum 2 years followup

**DOI:** 10.1051/sicotj/2016011

**Published:** 2016-05-13

**Authors:** Laurent Obert, Rachid Saadnia, François Loisel, Julien Uhring, Antoine Adam, Séverin Rochet, Pascal Clappaz, Tristan Lascar

**Affiliations:** 1 Orthopaedic, Traumatology and Hand Surgery Unit, Research Unit: EA 4268 I4S – IFR 133 INSERM, CHRU Besançon, University of Bourgogne Franche-Comté, Bd Fleming 25030 Besançon Cedex France; 2 Clinique Convert 01000 Bourg en Bresse France; 3 Orthopedic, Traumatology, and Hand Surgery Unit, Hopital Princesse Grace de Monaco Monaco Principauté de Monaco

**Keywords:** Shoulder fracture, Hemiarthroplasty, Locked stem, Tuberosities

## Abstract

*Introduction*: The purpose of this study was to evaluate the functional and radiological outcomes of a cementless, trauma-specific locked stem for 3- and 4-part proximal humeral fractures.

*Materials and methods*: This study consisted of two parts: a cadaver study with 22 shoulders and a multicenter prospective clinical study of 23 fracture patients evaluated at least 2 years after treatment. In the cadaver study, the locked stem (Humelock^TM^, FX Solutions) and its instrumentation were evaluated. In the clinical study, five senior surgeons at four different hospitals performed the surgical procedures. An independent surgeon evaluated the patients using clinical (Constant score, QuickDASH) and radiological (X-rays, CT scans) outcome measures.

*Results*: The cadaver study allowed us to validate the height landmarks relative to the pectoralis major tendon. In the clinical study, at the review, abduction was 95° (60–160), forward flexion was 108° (70–160), external rotation (elbow at body) was 34° (0–55), the QuickDASH was 31 (4.5–59), the overall Constant score was 54 (27–75), and the weighted Constant score was 76 (31.5–109).

*Discussion*: This preliminary study of hemiarthroplasty (HA) with a locked stem found results that were at least equivalent to published series. As all patients had at least a 2-year follow-up, integration of the locked stem did not cause any specific complications. These results suggest that it is possible to avoid using cement when hemiarthroplasty is performed for the humeral stem. This implant makes height adjustment and transosseous suturing of the tuberosities more reproducible.

## Introduction

Four-part fractures of the proximal humerus in patients between 60 and 70 years of age should theoretically be treated by hemiarthroplasty (HA). In this age group, use of reverse shoulder arthroplasty (RSA) implants is not the first line surgical solution and fracture fixation is not always feasible, either because of compromised humeral head vascularity (varus, extensive comminution, very osteoporotic bone) [1, 2] or because of a complex fracture pattern (varus, associated dislocation, low head volume). However, because the functional outcomes of HA for these fractures are correlated to implant height and anatomical reduction of the tuberosities [3–5], fewer and fewer surgeons are using this technique. For the same type of fracture, the results are more predictable with a RSA implant; this has contributed to a marked increase in the number of RSA procedures performed in patients between 60 and 70 years of age. We wanted to improve the functional outcomes after HA by developing a new trauma-specific implant. This implant was evaluated in a cadaver study and then in a small prospective clinical study.

## Materials and methods

**Figure 1.**
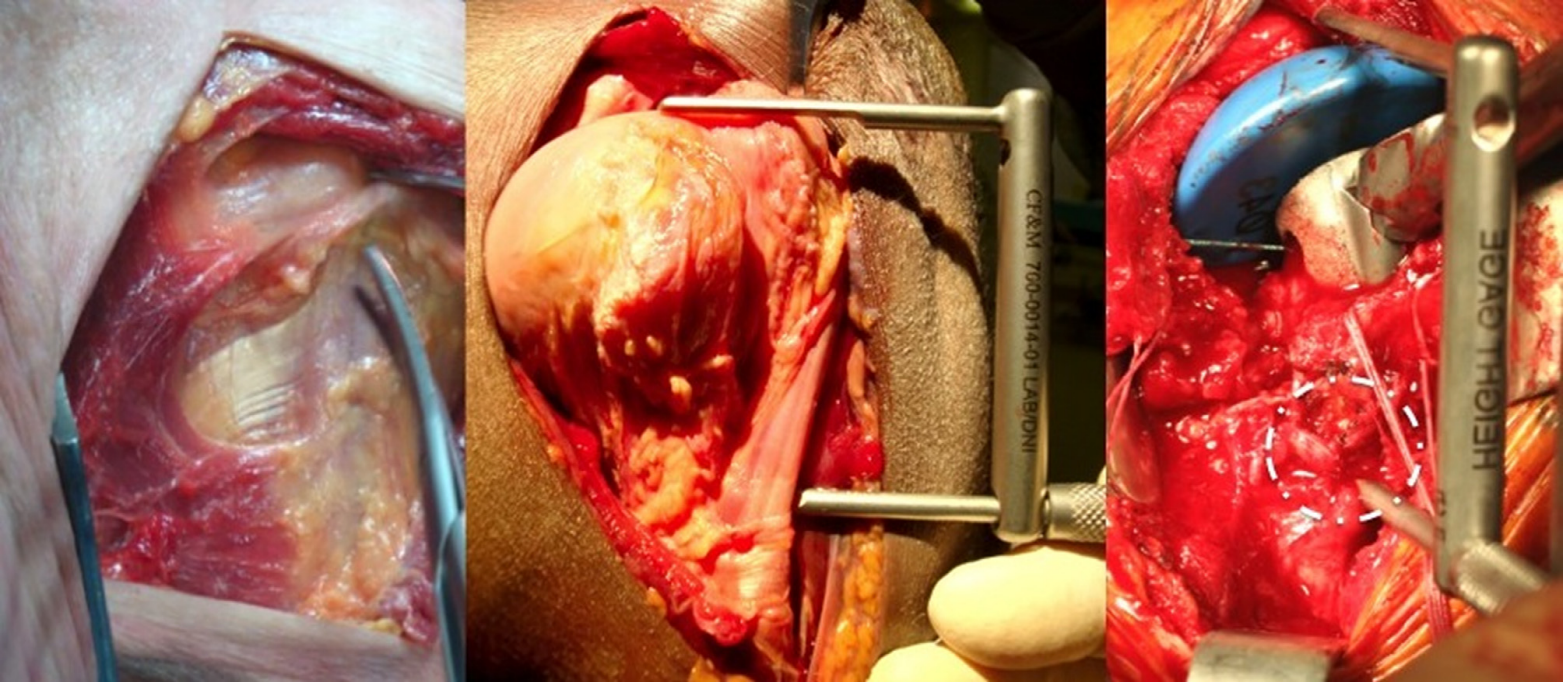
Three examples on left shoulders of the distance apex of the humeral head/upper edge of the pectoralis major: On the left, it is easy to find the upper edge of the pectoralis major on this anatomical dissection, in the center, one can see the scale of a specific measurement tool (with an arbitrary spread of 5.5 cm) measuring the distance between the apex of the humeral head/upper edge of the pectoralis major, on the right, a per-operative view of the positioning of the implant at a good distance from the upper edge of the pectoralis major (circled in white).

The first part of the study consisted of an evaluation of a fracture-specific stem with diaphyseal locking (Humelock^TM^, FX Solutions, Viriat, France) on 11 cadavers (22 shoulders). The goals of the cadaver study were to determine whether the diaphyseal locking mechanism was mechanically reliable, determine whether the implant was safe relative to the vascular and nerve structures, and verify the data generated by Murachowsky et al. [6] and Torrens et al. [7] on the distance between the upper margin of the pectoralis major tendon and the top of the humeral head (Figure 1).

To set the stem height, we decided to directly lock the chosen stem; this allowed us to focus our time on the aspect we felt was most important: fixation of the tuberosities. After locating the upper margin of the humeral head of the pectoralis major, the stem was locked in a trial position that placed the head 5.5 cm from the pectoral landmark. A K-wire was used to lock the distal end of the stem temporarily; this made it possible to carry out primary tuberosity reduction using trial heads and to check the configuration on fluoroscopy with the arm in neutral position, internal rotation, and external rotation.

Patients were recruited for the prospective clinical study conducted between 2009 and 2011. The patient inclusion criteria were adults treated by hemiarthroplasty for 3- and 4-part proximal humeral fractures with Humelock^TM^ justifying a 2-year follow-up. The exclusion criteria were patients treated by other prostheses than Humelock^TM^. Patients of each center were reviewed prospectively by an independent assessor. All patient consent was obtained.

Twenty-three patients had at least a 2-year follow-up at the time of the review. The clinical review consisted of measuring the shoulder motion range and calculating the Constant score and QuickDASH. The radiological review consisted of an analysis of the position and consolidation of the tuberosities around the implant; these elements were analyzed according to looped suture use. All patients received the fracture-specific Humelock^TM^ stem.

**Figure 2.**
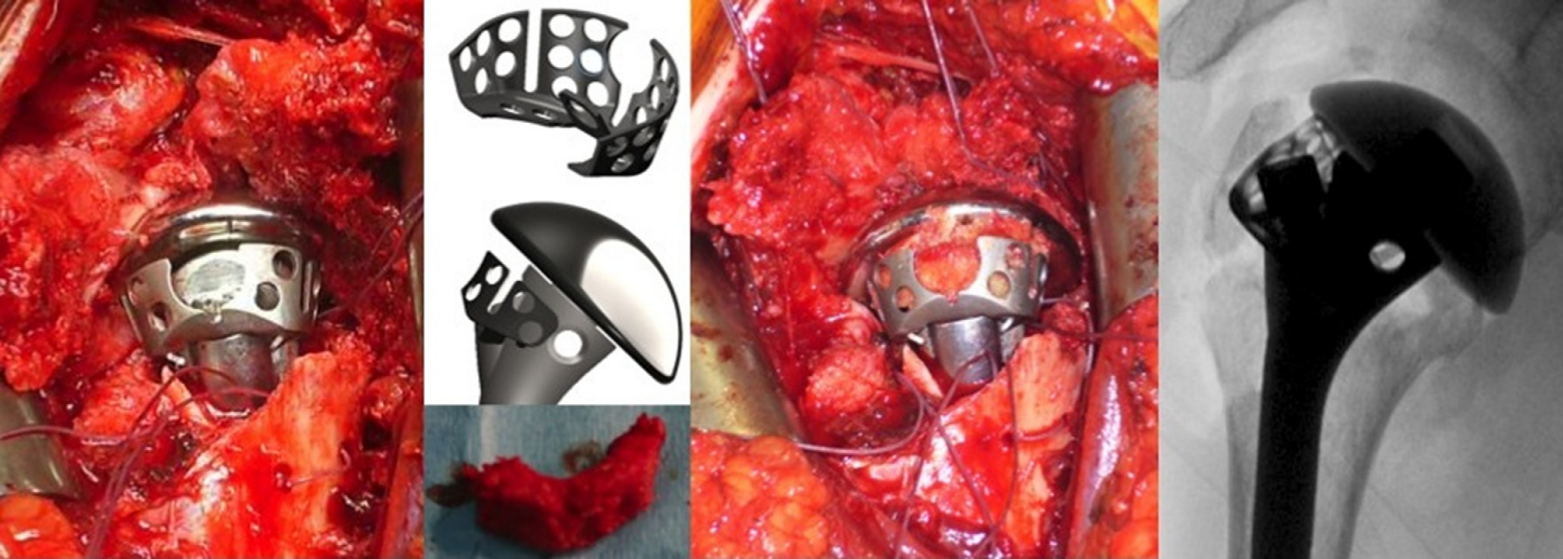
Looped thread system from left to right: The first to anchor and draw the tuberosities (in yellow), the next to press them to the implant passing through a hole designed for this (in blue), and the final group of two looped threads to create a vertical tie-down system (in green).

**Figure 3.**
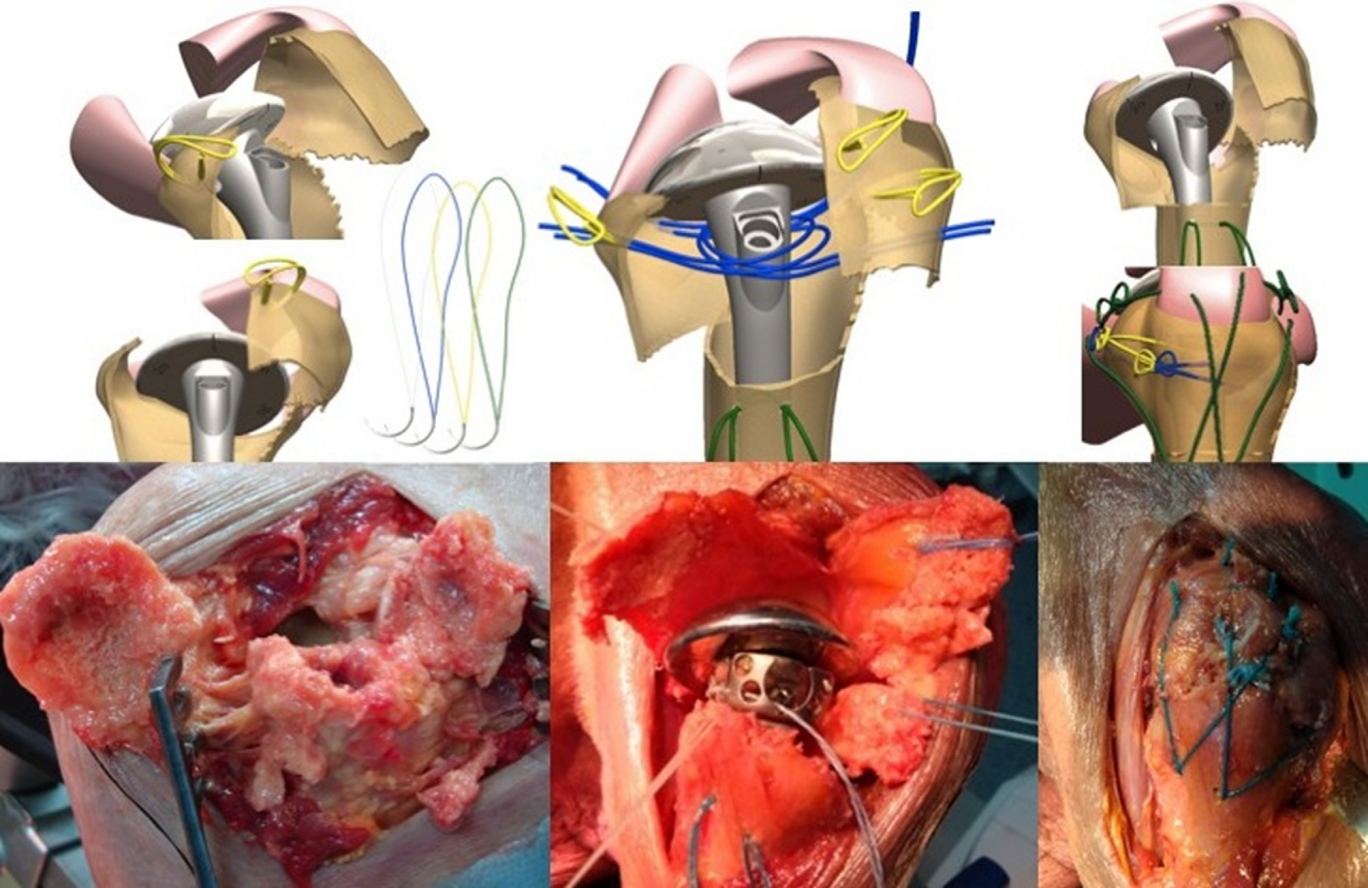
From left to right: The implant is positioned as well as the “cage” (Offset Modular System OMS^®^), an arched monobloc graft, sized to fit the humeral head, will be set inside the cage which is sufficiently soft to be molded, and sufficiently rigid to prevent medialization of the tuberosities.

This study also allowed us to evaluate tuberosity reattachment using specially-designed suture loops (Smartloop^®^, FX Solutions) (Figure 2), with the goal of generating a reliable and reproducible construct, independent of fracture type. We also evaluated the mechanical advantage of adding a cage (Offset Modular System^®^, OMS, FX Solutions) (Figure 3) below the head. This optional cage was developed to make it easier to position and fix the tuberosities as a function of the remaining tuberosity volume and to provide a recess where cancellous autograft taken from the patient’s humeral head can be added against the prosthesis. Twenty-one patients received the additional cage. The shoulder was immobilized in an internal rotation cast or sling for 4 weeks with no passive or active mobilization during this period. After four weeks, the patient began rehabilitation with active elevation and external 30° rotation. After eight weeks active external and internal rotations were possible. After 12 weeks, the patient was able to start working against resistance. Full resumption of activities was expected.

## Results

In the cadaver study, the distance between the top of the humeral head and the pectoralis major was 5.8 cm ± 5 mm, which is consistent with published data. This finding validated the height adjustment gauge developed for this implant. All stems could be locked solidly using the distal dual-locking instrumentation. The mechanical advantage of using the OMS to help position the tuberosities was evident in the cadaver study. The cage walls were further refined based on our findings to improve the biological exchanges around the implant.

**Figure 4.**
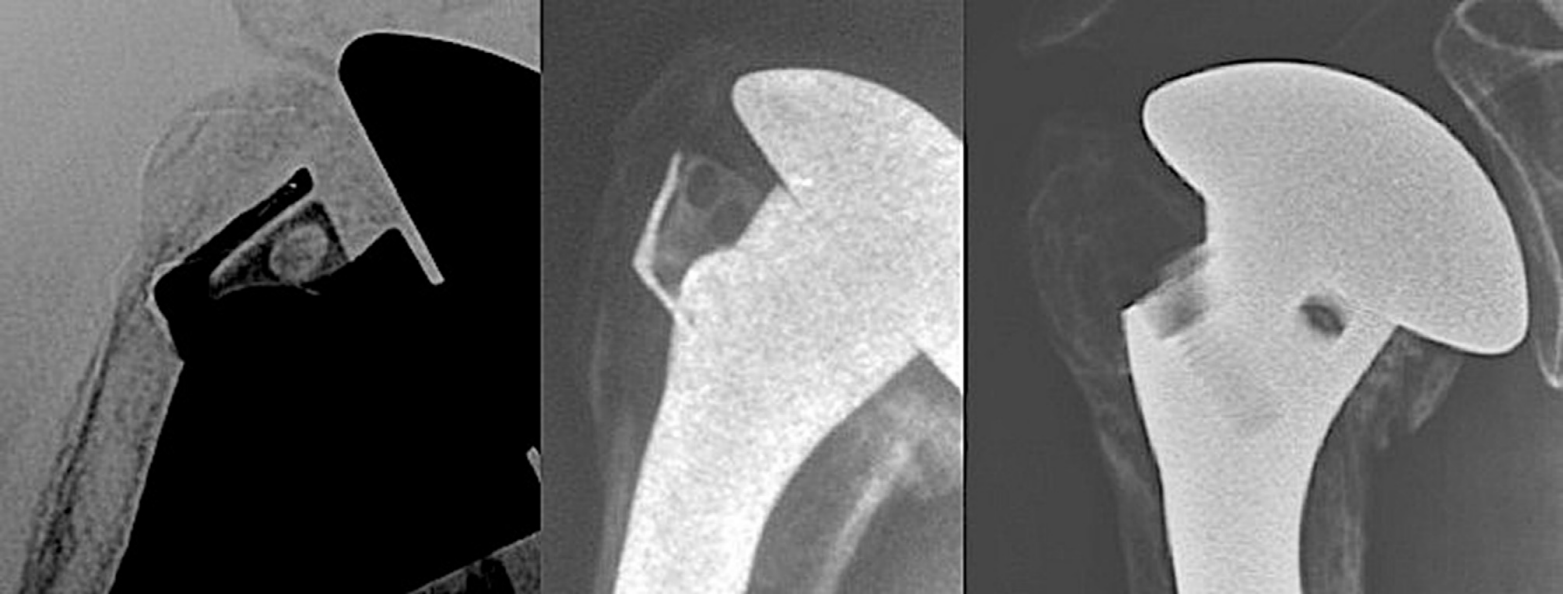
Three examples of tuberosity consolidation after 6 months obtained with the combination of looped thread grafts and OMS^®^. When tuberosities have a volume of bone which seems to be sufficient, OMS is not mandatory.

**Figure 5.**
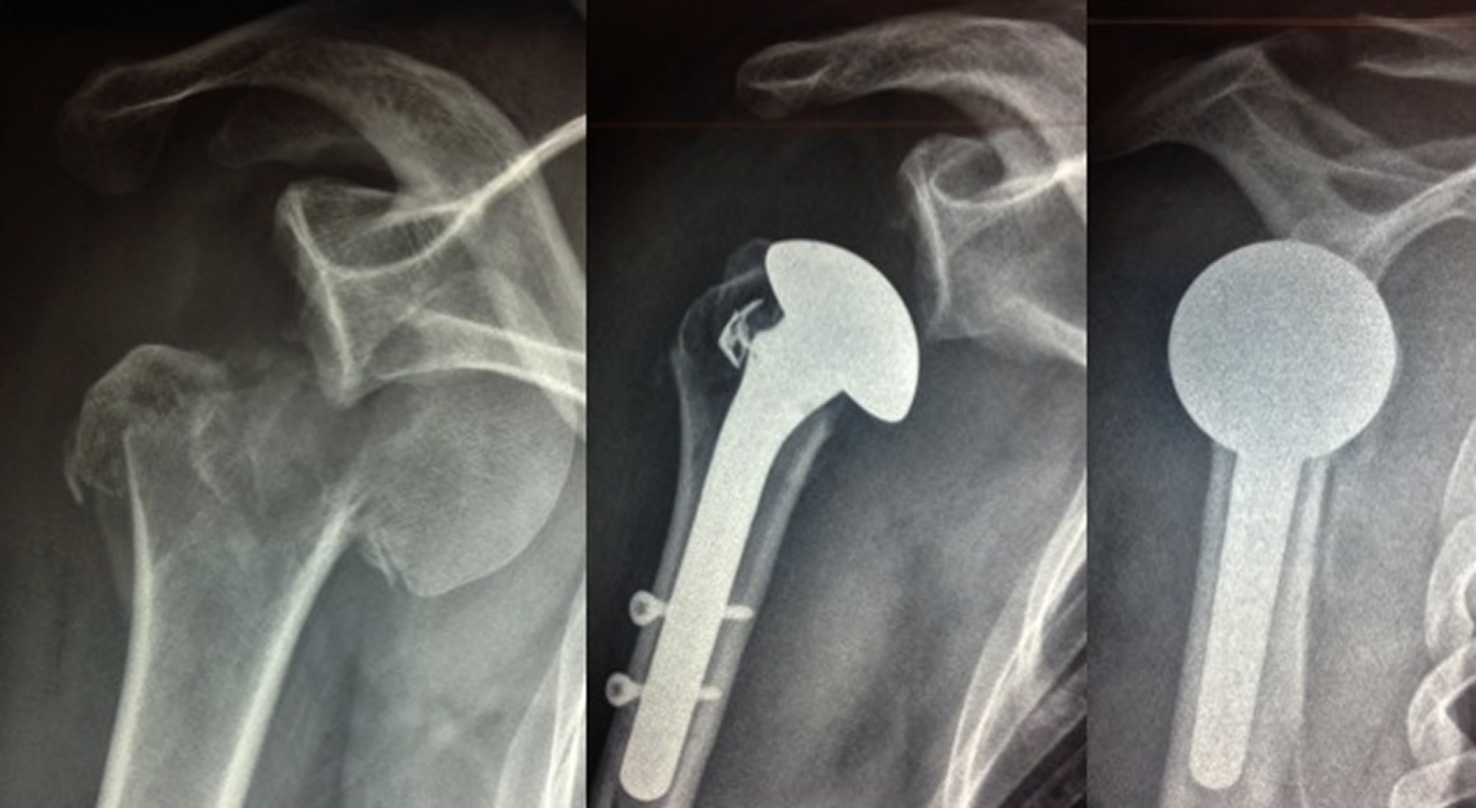
Four part dislocated fracture in a 55-year-old patient (right shoulder) and postoperative X-ray.

**Figure 6.**
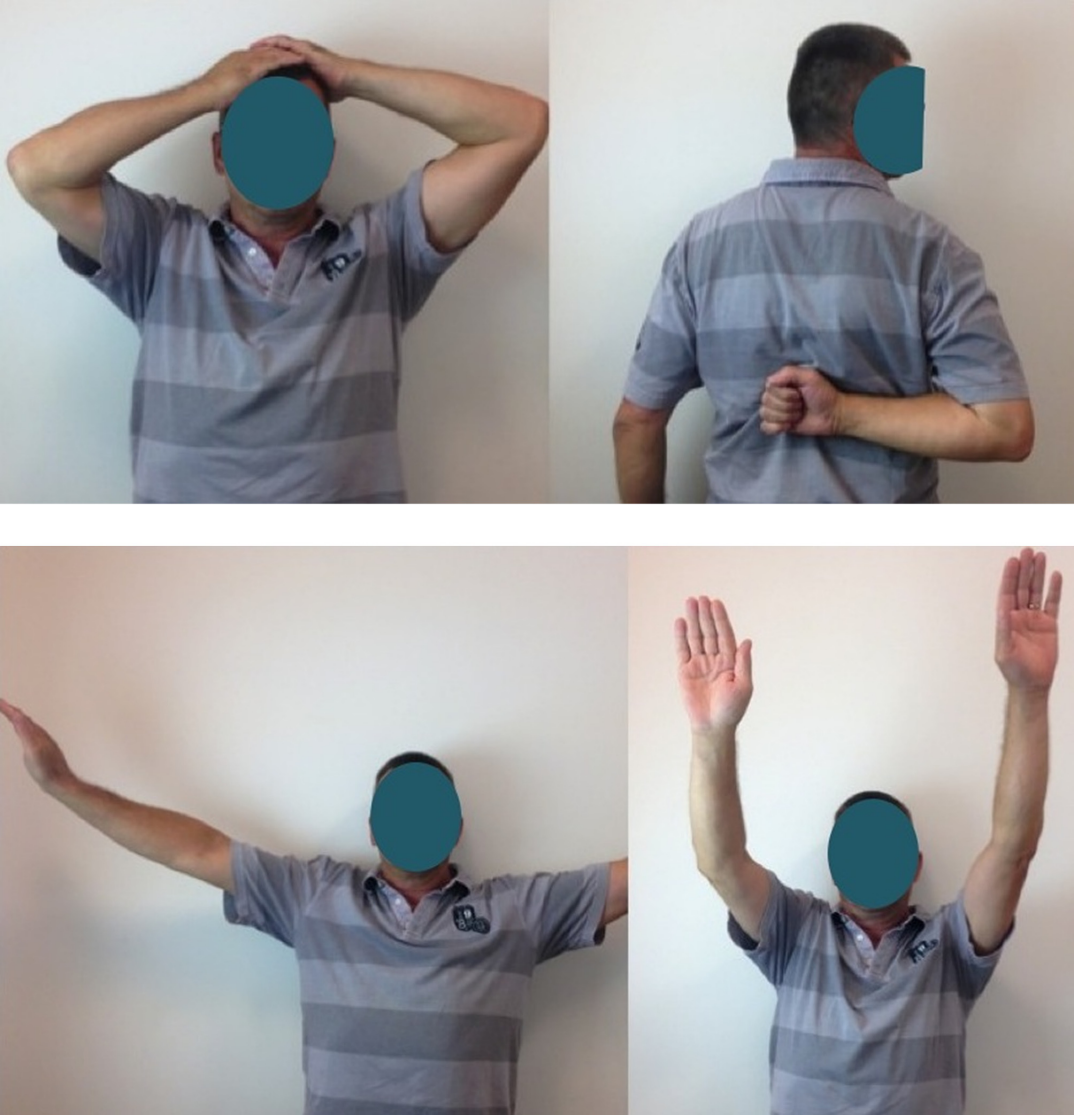
Functional results at 26 months of follow-up with a Constant score = 81 (90 if adjusted).

**Figure 7.**
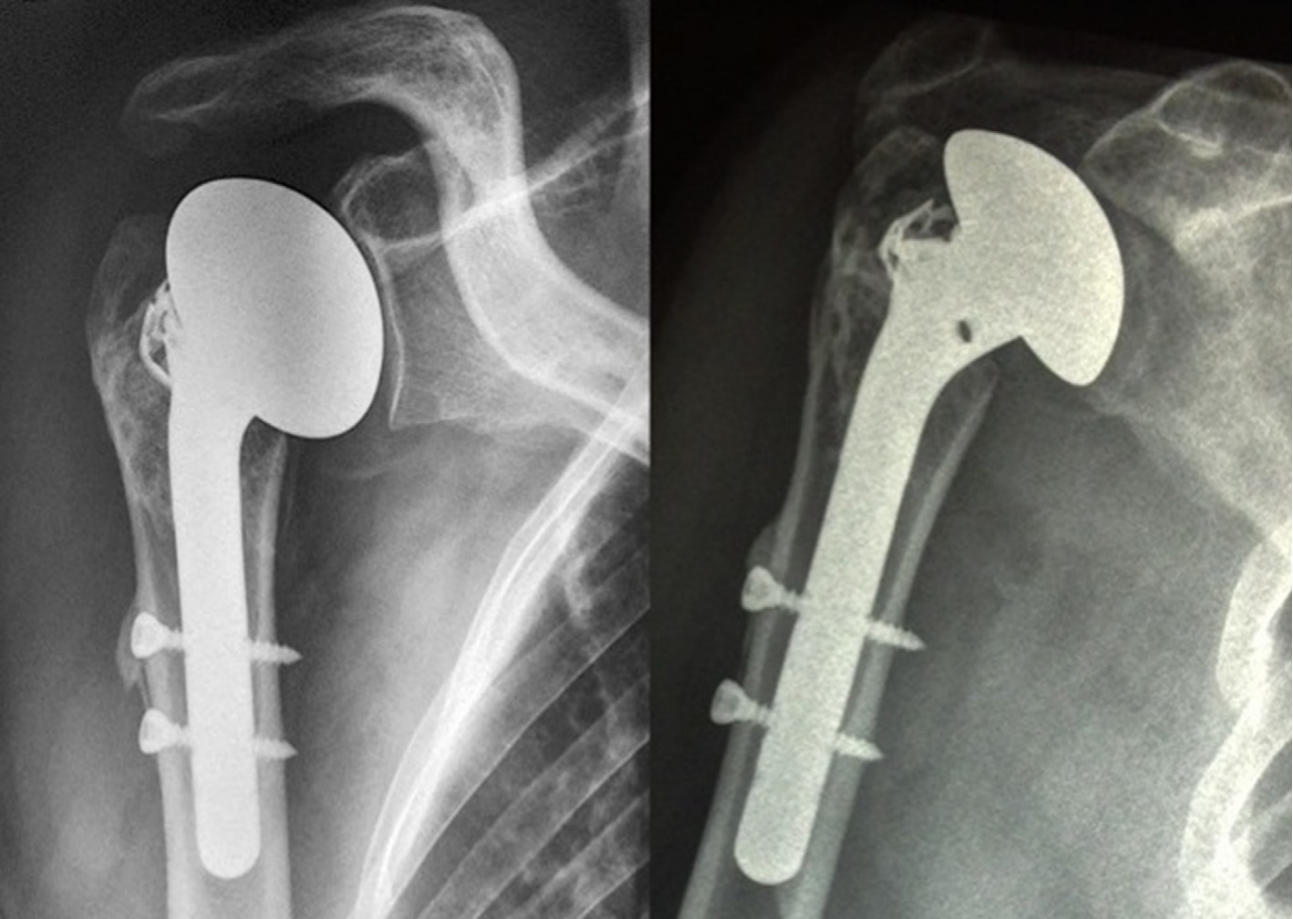
Bone union on X-ray at same follow-up.

The clinical study enrolled 23 patients (4 men, 19 women). The average patient age was 67.3 years (50–90). Five senior surgeons at four different hospitals performed the surgical procedures. The patients were reviewed at a mean of 51.3 months (24–96). There were 19 four-part fractures and four–three-part fractures. According to Duparc classification system for four-part fractures, one fracture was CT2 (impacted in valgus), 12 were CT3 (all fragments displaced), and five were CT4 (head dislocated). Eighteen cases involved the dominant side. There was no associated axillary nerve palsy. The patients were operated a mean of 7.1 days (1–17) after the fracture event by deltopectoral approach. At each patient’s maximum follow-up, the average abduction was 95° (60–160), forward flexion was 108° (70–160), external rotation with elbow at side was 34° (0–55), internal rotation reached L3, the QuickDASH was 31 (4.5–59), the overall Constant score was 54 (27–75), and the weighted Constant score was 76 (31.5–109). Forward flexion, abduction, weighted, and global Constant scores were significantly better when the greater tuberosity healed in its anatomical position (Figures 4–7). The QuickDASH improved but the change was not significant. There were 11/23 cases (47%) with excellent or good results (weighted Constant score > 80%) and 7/23 cases (30%) with bad results (weighted Constant score < 70%). No infections or dislocations were observed at the time of the last follow-up. In two patients in whom the tuberosities were not reduced postoperatively, the shoulder was stiff with nonunion evident at the lesser tuberosity.

Five complications occurred: one intraoperative fracture requiring cerclage wire, two cases of capsulitis, and two cases of rotator cuff damage after 14 months in both cases. One of these rotator cuff cases required RSA conversion, which was easy to perform because no cement had been used initially. No complications related to stem locking were observed.

## Discussion (Tables 1 and 2)

Consolidation of the lesser tuberosity and retroversion of the humeral stem are difficult to evaluate reliably. CT scanning would have provided a more accurate evaluation, but would also have exposed the patient to more radiation. It was not possible to determine whether the humeral length had been restored because X-rays of the entire humerus were not taken.

**Table 1.** Hemiarthroplasty review of literature.

Series No. patients, age, % women, followup (months)	Fracture type, approach, tub suturing	Global Constant (GC), weighted Constant (WC), DASH	Tuberosity union	Clinical complications	Radiol. complications	Comments
Goldman (9), *n* = 22, age = 68 14 F (63.6%), F/U = 30	3-part fracture: 10, 4-part fracture: 12, deltopectoral all cases	N/R	100% anatomical tuberosity union	No infections, No nerve injuries 1 wound dehiscence	3 proximal implant migration (13.6%) - 7 humeral loosening (31.8%) - 3 ectopic ossification (13.6%)	Being female, having a 4-part fracture and being > 70 y/o were predictors of negative joint range of motion results
Boileau (7), *n* = 66, age = 66, 45 F (68%), F/U = 27	4-part fracture: 59, 3-part fracture: 7, deltopectoral all cases	GC: 56, WC: 74%	33 malpositioned (50%), 11 nonunion (17%), 26 malunion (39%)	- 3 transient axillary nerve damage (4.5%) - 1 anterior dislocation (1.5%)	- 7 ectopic ossification (10.5%) - 15 proximal implant migration (22%) - no loosening - 16 radiolucent lines < 1 mm (24%)	- Wrong humeral stem height (>10 mm lengthening or >15 mm shortening) or retroversion (> 40°) is correlated with poor functional results and incorrect tuberosity positioning - Final malpositioning of the tuberosities is correlated with a poor functional result - Being female and being > 75 y/o are significantly correlated with poor functional results and tuberosity migration
Prakash (30), *n* = 22, age = 69, 19 F (86%), F/U = 33	4-part fracture: 12, 4-part dislocated fracture: 3, 3-part fracture: 7, deltopectoral approach in all cases	N/R	- 2/16 lesser tuberosity malunion (12.5%) - 1/16 lesser trochanter nonunion (6.2%) - 13/16 anatomical lesser tuberosity union (81.2%)	- 1 anterior dislocation (4.5%)	- 1 aseptic loosening at 7 years F/U (surgical revision) (4.5%) - 1 ectopic ossification (4.5%) - 1 anterior subluxation (4.5%)	Range of motion was significantly better in patients 65 years of age or younger
Mighell (11), *n* = 72, age = 66, 54 F (76%), F/U = 36	4-part fracture: 41, 3-part fracture: 22, intra-articular fracture: 8, anatomical neck fracture: 1, deltopectoral approach in all cases	ASES: 76.6 (25–100)	- 69 tuberosity union (96%) - 54 anatomical tuberosity union (75%) - 15 malunion (21%) - 3 nonunion (4%)	- 1 deep infection (1.4%) - 1 CRPS (1.4%)	- 15 proximal implant migration (20.8%) - 1 aseptic loosening (1.4%) (surgical revision) - -1 septic loosening (1.4%) - - 18 ectopic ossification (25%)	Proximal implant migration is correlated with poor functional results
Kralinger (15), *n* = 167, age = 70, 127 F (76%), F/U = 29	3-part fracture: 17, 4-part fracture: 109, fracture-dislocation: 41, Not specified	GC: 55.4	- 28 (16.8%) union with > 0.5 cm displacement - 62 (37.1%) union with < 0.5 cm displacement - 77 (46.1%): osteolysis, nonunion, malunion	- 1 superficial infection (0.6%) - 1 deep infection (0.6%)	- 3 anterosuperior subluxation (1.8%)	- Tuberosity union is significantly affected by age but not by bone graft use - Anatomical tuberosity union positively affects the Constant score
Gronhagen (6), *n* = 46, age = 69, 37 F (80.4%), F/U = 53	2-part fracture: 2, 3-part fracture: 10, 4-part fracture: 34, deltopectoral approach in all cases	GC: 42	- 5 secondary displacement	- 1 superficial infection (2%) - 1 dislocation (2%)	- 24 proximal implant migration (52%) - 16 glenoid erosion (35%) - 25 ectopic ossification (54%) - no loosening	- Constant score is significantly higher in patients under 60 years of age. - Fracture types does not significantly affect Constant score - Constant score is significantly better in the "no migration" group than the implant migration group
Antuna (16), *n* = 57, age = 66, 44 F (77%), F/U = 126	4-part fracture: 32, 3-part fracture: 7, 4-part dislocated fracture: 9, 3-part dislocated, fracture: 4 intra-articular fracture: 5, deltopectoral approach in all cases	N/R	22/35 anatomical tuberosity union (62.8%)	- 1 early posterior dislocation (1.7%)	85% subluxation: - 18 superior - 6 anterior - 2 posterior - 13 humeral radiolucent lines - 1 stem loosening (surgical revision)	Anatomical tuberosity union and being under 70 years of age are significantly correlated with better forward flexion
Kontakis (17), *n* = 28, age = 66.4, 23 F (82%), F/U = 39.3	4-part fracture: 18, 3-part fracture: 2, 4-part dislocated fracture: 4, 3-part dislocated fracture: 4, deltopectoral approach in all cases	GC: 68.2	13 anatomical reduction 14 acceptable reduction 1 initial malpositioning - 0 nonunion	No dislocation, infection, nerve damage or instability	- 5 proximal implant migration (17.8%) - no loosening - no radiolucent lines	Anatomical tuberosity union leads to non- statistically significant improvements in Constant score and ROM (*P* > 0.05)
Esen (18), *n* = 42, age = 68.9, 28 F (67%), F/U = 78.8	4-part fracture: 25, 3-part fracture: 7, 3-part dislocated fracture: 6, intra-articular fracture: 4, deltopectoral approach in all cases	GC: 73.6	- 3 osteolysis - 37 anatomical tuberosity union (88%) - 0 nonunion	- 2 transient axillary nerve damage (4.8%) - 1 CRPS (2.4%) - 1 postoperative hematoma with surgical revision	- 2 proximal implant migration (4.8%) (surgical revision) - 9 RLL around stem (30%) - no stem loosening	- Anatomical tuberosity union significantly improves forward flexion - Positive correlation between humeral offset and flexion ROM - Positive correlation between acromiohumeral height and flexion ROM - Negative correlation between implant height and flexion ROM
Reuther (5), *n* = 102, age = 71.5, 88 F (86%), F/U = 28.1	4-part fracture: 60.9%, 3-part fracture: 20.7%, intra-articular fracture, other: 18.4%, deltopectoral approach in all cases	GC: 44.7 - pain: 10.4/15 - activity: 12.3/20 - mobility 13.7/40 - strength: 8.3/25 - WC: 62.8% ASES: 61.5	- 36 anatomical tuberosity union (35.3%) - 66 malunion or nonunion (64.7%)	N/R	N/R	- Anatomical tuberosity union significantly improves the Constant score and ASES score - Being female, having osteoporosis and being older are predictors of tuberosity nonunion - No significant differences in tuberosity union when bone graft used
Shah (27), *n* = 32, age = 72.2, 24 F (75%), F/U = 25.3	4-part fracture: 21, 4-part dislocated fracture: 7, 3-part fracture: 3, deltopectoral approach in all cases	ASES: 67.2, UCLA: 24.8	- 31 tuberosity union (97%) - 1 lesser trochanter nonunion (3%)	- 1 superficial infection (3%) - 1 transient axillary nerve damage (3%)	- 10 proximal implant migration (31%) - 1 anterior subluxation - no RLL	Functional outcomes are significantly affected by the preoperative condition of the rotator cuff, but also by age, gender and proximal implant migration
Padua (36), *n* = 50, age = 73, 38 F (76%), F/U = 38.4	Fracture type not recorded, deltopectoral approach in all cases	ASES: 56.85, DASH: 39.29	N/R	N/R	N/R	No correlation between implant height and functional scores (DASH, ASES) No correlation between joint range of motion and implant height or retroversion Significant correlation between retroversion and functional scores (DASH, ASES)
Castricini (34), *n* = 57, age = 72.2, 53 F (93%), F/U = 52	3-part fracture: 7 (12%), 4-part fracture: 42 (73%), fracture- dislocation: 8 (14%), deltopectoral approach in all cases	GC: 59.2 - pain: 14/15 - mobility: 25.3/40 - activity: 16.3/20 - strength: 3.3/25	- 41 anatomical lesser tuberosity union (73.2%) - 9 lesser tuberosity malunion (16.1%) - 6 lesser tuberosity osteolysis (10.7%)	No infections - No nerve injuries - No dislocations	- 7 proximal implant migration (12.5%) - 5 ectopic ossification (8.9%) - No loosening or radiolucent lines	Better Constant scores achieved in patients with anatomical tuberosity union and no proximal implant migration
Fucentese (37), *n* = 30, age = 63.3, 10 F (33.3%), F/U = 25	3-part fracture: 3 (10%), 3-part dislocated fracture: 4 (13.3%), 4-part fracture: 23 (76.6%), deltopectoral approach in all cases	GC: 59, WC: 75%	- 23 anatomical lesser tuberosity union (85%) - 4 secondary lesser tuberosity displacement (2 surgical revisions, RSA conversion)	No infections - No nerve injuries - No dislocations	- 3 ectopic ossification - 20 lesser trochanter osteolysis (12 severe, 8 medium) - no loosening	Large metaphysis implant that results in good rate of anatomical tuberosity union, but no control group included
Boileau (10), *n* = 60 (61 shoulders), age = 67, 38 F (63.3%), F/U = 64 group A: standard stem (*n* = 31), group B: fracture-specific stem (*n* = 30)	4-part fracture: 56 (92%), 3-part fracture: 5 (8%), deltopectoral approach in all cases	GC: - all patients: 63 - group A: 58.9 - group B: 68.2 WC: all patients: 89% - group A: 84% - group B: 93%	**Anatomical lesser tuberosity union:** - all patients: *n* = 40 (66%) - group A: *n* = 14 (45%) - group B: *n* = 26 (87%) **Lesser tuberosity malunion:** - all patients: *n* = 17 (27.9%) - group A: *n* = 14 (45%) - group B: *n* = 3 (10%) - **Lesser tuberositie nonunion:** - all patients: *n* = 4 (6.5%) - group A: *n* = 3 (9.7%) - group B: *n* = 1 (3.3%)	- 1 axillary artery damage (1.6%) - 1 deep infection (1.6%) - 2 transient axillary nerve damage (3.3%) - 2 capsulitis (3.3%)	- 2 glenoid erosion (3.3%) (surgical revision)	**Study comparing standard humeral stem to fracture-specific stem** - Use of fracture-specific stem led to significant improvements in: - anatomical lesser **tuberosity** union rate - all parameters of the Constant score, except pain and internal rotation Being 75 y/o or greater, being female and using a standard stem are risk factors for tuberosity nonunion and poor functional outcomes Anatomical lesser tuberosity union significantly improves the Constant score
Brandao B (38), *n* = 67, age = 65, 47 F (70%), F/U = 38	4-part fracture: 46, 3-part fracture: 18, deltopectoral approach in all cases	UCLA: 26	33 anatomical union of lesser tuberositie (49%)	- 1 periprosthetic fracture intraoperative - 1 periprosthetic fracture at 11 months followup - 2 recessive nerve damage (median and axillary nerves) - 1 deep infection (1.5%)	N/R	Anatomical tuberosity union significantly improves the functional outcomes Men had significantly better forward flexion and UCLA scores than women

**Table 2.** Joint range of motion reported in published studies of hemiarthroplasty for proximal humerus fractures.

		No. of patients reviewed	Age (years)	Followup (months)	Forward flexion	Abduction	Ext Rot 1	Int Rot 1
Hemiarthroplasty	Goldman (9)	22	68	30	107°	–	31°	L2
	Boileau (7)	66	66	27	101°	–	17.5°	L3
	Prakash (30)	22	69	33	93°	–	23°	L1
	Christoforakis (31)	16	62.7	45.7	150°	145°	30°	L3
	Mighell (11)	72	66	36	128°	–	43°	L2
	Kralinger (15)	167	70	29	41.9% > 90°	–	–	–
	Jacquot (19)	72	69	18	130°	–	–	–
	Krishnan (24)	32	72	18	117°	–	–	–
	Loew (32)	39	72	29.3	91.8°	88.1°	17.2°	–
	Padua (33)	21	70	41	113°	88°	46°	L2
	Antuna (16)	57	66	126	100°	–	30°	L5
	Gallinet (26)	17	74	16.5	53.5°	60°	13.5°	–
	Kontakis (17)	28	66.4	39.3	149°	144°	26.2°	–
	Esen (18)	42	68.9	78.8	121°	–	30	L5
	Reuther (5)	102	71.5	28.1	62.6°	60°	–	–
	Shah (27)	32	72.2	25.3	85.1°	–	–	–
	Castricini (34)	56	72.2	52	106°	–	19°	L3
	Liu (35)	33	64.3	44.4	102°	–	31°	L5
	Padua (36)	50	73	38.4	95.7°	82.1°	21.4°	L2
	Fucentense (37)	29	63.3	25	117°	111°	–	–
	Boileau (10)	60	67	64	124.8°	–	29°	L3
	Brandao (38)	67	65	38	104°	–	36°	L1
	**Current study**	**23**	**67.3**	**51**	**108°**	**95°**	**34°**	**L3**

The number of patients in this study, as well as their ages and genders, were comparable to other published studies. The effect of age on tuberosity consolidation, and in parallel, the functional outcomes, has been demonstrated by several authors. Reuther et al. [3] found that the tuberosity union rate was 61.5% in patients under 60 years of age, but only 26.5% in those over 80. Grönhagen et al. [4] showed that the Constant-Murley score was significantly better in patients under 60 years of age. Boileau et al. [5] found that tuberosity migration was significantly correlated to being over 75 years of age. In the current study, no differences were observed between patients over and under 60 years of age. Comorbidities also impact the functional outcomes. Kabir et al. [8] found that the Constant-Murley score decreased from 41 to 27 when patients have three or more comorbidities.

Many studies have shown that women have worse functional outcomes and joint range of motion [5, 9, 10]. This can be explained by poor bone quality and increased risk of tuberosity nonunion. However, we found no differences in the outcomes between genders in the current study.

We did not find the time allowed before surgery to have any impact on clinical outcomes. Mighell et al. [11] reported that functional outcomes were significantly better when patients were operated within 2 weeks of the fracture event; this concept was not supported by Fallatah’s study [12]. The deltopectoral approach used in this study is the same approach used in most published studies. A significant correlation between stem height and consolidation of the tuberosities in their anatomical position has been found [5]. A reliable reference for appropriate stem height is the distance between the apex of the humeral head and the superior margin of the pectoralis major tendon [6]. This distance can be measured only when using the deltopectoral approach. Better clinical outcomes have been found with the height they set using the distance between the apex of the humeral head and the superior margin of the pectoralis major tendon [13, 14]. In our opinion, bone grafting is essential because it increases the primary stability of the tuberosities and adds a biological element to the healing process. We believe that the HA results are more predictable when the tuberosities are stabilized by transosseous sutures placed around the implant’s metaphysis, which is filled with autograft. A survey in this field will again be investigated.

Some authors have shown that tuberosity consolidation is not significantly affected by the use of bone graft [3, 15]. However, several teams have shown that tuberosity consolidation is critical to achieve good functional outcomes [3, 5, 15–18]. Complications related to tuberosity consolidation are the main cause of functional catastrophe.

The nonunion rate in the current study (11.5%) falls within the range (0%–17%) reported in other studies [5, 9–11, 15, 17–20]. Transosseous tuberosity suturing must be performed meticulously. Anatomical reduction of the tuberosities is an essential prerequisite for good functional outcomes. The malunion rate in the current study (23.7%) falls within the range (0%–39%) reported in other studies [5, 9].

Other studies found a tuberosity nonunion or malpositioning rate of 40%–66% [21–23]. The transosseous sutures are as important as the implant’s design and height for ensuring that the tuberosities heal in the correct position. Krishnan et al. [24] and Boileau et al. [25] have recently reported tuberosity nonunion rates of 21% and 13%, respectively, when a fracture-specific stem was used. This reinforced our pursuit of implant that effectively treats humeral fractures involving the head and tuberosities.

The intraoperative complication rate in this study was low. The published rate is under 2%. Boileau et al. [10] reported one case of axillary artery damage (1.6% complication rate). Brandão et al. [26] reported one periprosthetic humeral fracture (1.5% rate).

All the patients in this study underwent postoperative immobilization for one month using a shoulder immobilizer with the arm internally rotated. This position is controversial. Some authors advocate immobilization in neutral position or external rotation to reduce tension on the lesser trochanter, as this is a source of migration [5, 27]. However, this position is difficult to maintain during sleep.

Although Robinson et al. [28] have shown that the Constant score levels out starting at the sixth postoperative month; very few studies have more than two years follow-up. An analysis of published results shows a wide variation in the resulting shoulder range of motion (ROM). The mean forward flexion ranges from a low of 53.5° [29] to a high of 149° [17]. The Constant-Murley score ranges from a low of 42 points [4] to a high of 73.6 points [18]. The overall Constant-Murley score of 45.9 in this study falls within this range.

The condition of the rotator cuff directly affects the functional results of HA for fracture. Impaired functional outcomes due to reduced subacromial space have been demonstrated in many studies [4, 9, 11] and can reach 52% [4].

At the latest review, the functional outcomes were significantly altered when the subacromial space was less than 7 mm (34.6%). In this study, it can be attributed to secondary rotator cuff damage in all cases. No damage to the rotator cuff was identified intraoperatively. One cause of postoperative rotator cuff damage is excessive humeral length. According to Boileau et al. [5], lengthening of more than 10 mm had two effects:

- rotator cuff damage (less subacromial space) due to excessive tension on the supraspinatus muscle;
- tuberosity migration due to lack of union with the humeral shaft.

Humerus length could not be determined in the current study because X-rays of the entire humerus were not taken.

Mighell et al. [11] found a smaller subacromial space in 20.8% of patients. This radiological finding is systematically associated with impaired functional outcomes. Shah et al. [19] counter these results and found no significant difference between patients who have reduced subacromial space and those who did not.

No infections were identified in this study, which has a minimum follow-up of two years. The published rate of deep infection after HA for fracture is 1.6% [4, 10, 11, 15, 19, 26]. For all etiologies combined, the prevalence of infection of anatomical shoulder implants is 2% according to Pelegri et al. [30]. Staphylococci and Propionibacterium acnes are the microorganisms most commonly found (23%–40%). The infectious prognosis is relatively good: the infection is resolved in 71% cases when all treatments are considered together.

Ectopic bone formation identified on X-rays is generally not considered as a complication. Grönhagen et al. have reported ectopic bone formation in 54% of cases [4]. The impact of this abnormal radiological finding on functional outcomes is controversial. Grönhagen et al. [4] and Goldman et al. [9] found no changes in the functional outcomes in patients with ectopic ossification. Kjaergaard-Andersen et al. [31] described three stages of ectopic ossification in a series of 26 out of 58 arthroplasty cases. In stage III, the bone bridges the humerus and acromion. The functional outcome is negatively affected only after ossification reaches stage III.

Other authors have published series on hemiarthroplasty for proximal humerus fractures. Their results are reported in Table 1: Prakash et al. [20], Christoforakis et al. [32], Loew et al. [33], Padua et al. [34], Castricini et al. [35], Liu et al. [36], Padua et al. [37], Fucentese et al. [38], Brandão et al. [26].

## Conclusion

This is a relatively short-term study, but optimization of a fracture-specific HA implant leads to more predictable results – if the tuberosities are reduced they will heal. The predictable results with this implant can be explained by the ease of setting the stem height, the ability to lock the stem in place without cement, the use of suture loops for transosseous fixation, and the stabilization of the horseshoe-shaped graft in the metaphyseal area. In the hands of an experienced surgeon who makes use of fluoroscopy, the results of HA can be improved without having to perform RSA on every fracture case.

## Conflict of interest

Authors LO, PC, and TL have or may receive payments or benefits from FX Solutions related to this work. The other authors certify that they have no financial conflict of interest.
